# Hepatoid adenocarcinoma of the stomach with PIK3Ca mutation during pregnancy: A case report with molecular profile

**DOI:** 10.1093/omcr/omab078

**Published:** 2021-09-13

**Authors:** Anastasija Ranceva, Rokas Stulpinas, Rimvydas Norvilas, Ugnius Mickys

**Affiliations:** 1 Hematology, Oncology and Transfusion Medicine Center, Vilnius University Hospital Santaros Klinikos, Vilnius, Lithuania; 2 National Center of Pathology, Affiliate of Vilnius University Hospital Santaros Klinikos, Vilnius, Lithuania

**Keywords:** hepatoid adenocarcinoma, pregnancy, gastric cancer, PIK3CA, NGS

## Abstract

Hepatoid adenocarcinoma is an extremely aggressive special subtype of gastric tumors. It can be lethal as no standard treatment options for this type of gastric cancer exist. Here, we describe a very rare case of a young female on her 21^st^ week of pregnancy who was diagnosed with stage IV hepatoid adenocarcinoma of the stomach with elevated α fetoprotein (AFP) level. Gene mutation analysis performed by next-generation sequencing identified somatic mutations in the *PIK3CA* gene. Despite the treatment, patient died 2 months after the initial disease presentation. To our best knowledge, this case represents the first report of pregnancy-associated hepatoid gastric adenocarcinoma with the *PIK3CA* gene mutations, which can provide further clues for the understanding of molecular features of this type of tumor that can reflect biological behavior and may lead to further effective treatment options.

## INTRODUCTION

The incidence of gastric cancer during pregnancy is low and attributed to 0.1% of all cases. It is not only rare, but also difficult to diagnose as the symptoms may be regarded as features of an ordinary pregnancy. When combined with an aggressive histological subtype such as hepatoid adenocarcinoma of the stomach (HAS) it can be lethal because is found to have a poor prognosis with no standard treatment options in comparison to other types of gastric cancer [1–4].

HAS represents a unique subtype of gastric cancer with specific clinicopathological characteristics with evidence of α fetoprotein (AFP) production and histologic features mimicking hepatocellular carcinoma. It has been associated with liver and lymph nodes metastasis and aggressive clinical course. [5, 6]. The median survival of HAS patients is about 12 months [7–9].

The exact molecular mechanism of HAS remains unclear. Specific driver gene mutations and biomarkers in HAS are unknown. No standard therapies for HAS are recommended currently [10].

In this report we present a rare case of hepatoid gastric cancer in a young pregnant female, who was diagnosed accidentally with stage IV HAS with elevated AFP level. The gene mutation analysis performed by next-generation sequencing (NGS) identified mutations in the *PIK3CA* gene that might be associated with tumor development.

## CASE PRESENTATION

**Figure 1:**
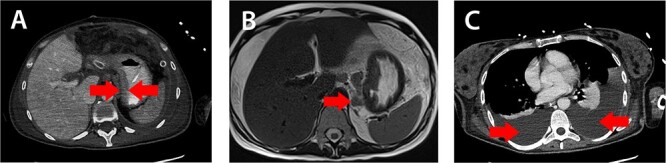
Magnetic resonance imaging: A) thickening of the gastric wall, B) perigastric lymph node enlargement and C) pleural effusion (red arrows).

**Figure 2:**
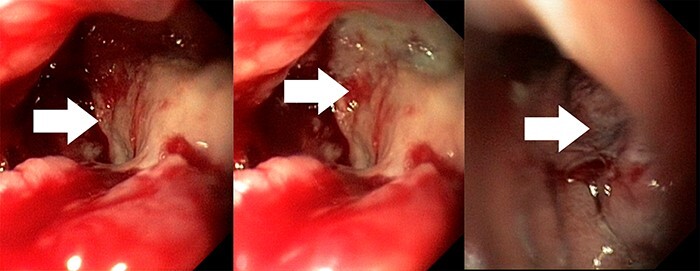
Esophagogastroduodenoscopy imaging: an infiltrative ulcerated tumor of the stomach 4 cm in size (white arrows).

A 38-year-old female was admitted to the hospital on the 21st week of her first pregnancy complaining of abdominal pain, nausea, vomiting, diarrhea, and weight loss. The patient had no history of liver disease and gastrointestinal problems before. Laboratory findings revealed anemia and elevated C-reactive protein. Ultrasonography of the abdomen showed the presence of ascites, hydronephrosis (II°) and a fluid in the pleural cavities. Magnetic resonance imaging revealed thickening of the gastric wall, enlarged perigastric lymph nodes and ascites (Figure 1). Esophagogastroduodenoscopy showed an infiltrative ulcerated tumor in the lesser curvature of the stomach ~ 4 cm in size (Figure 2). Serum AFP level was elevated to 79,6 kU/l (normal range 0,5–5,5kU/l).

**Figure 3:**
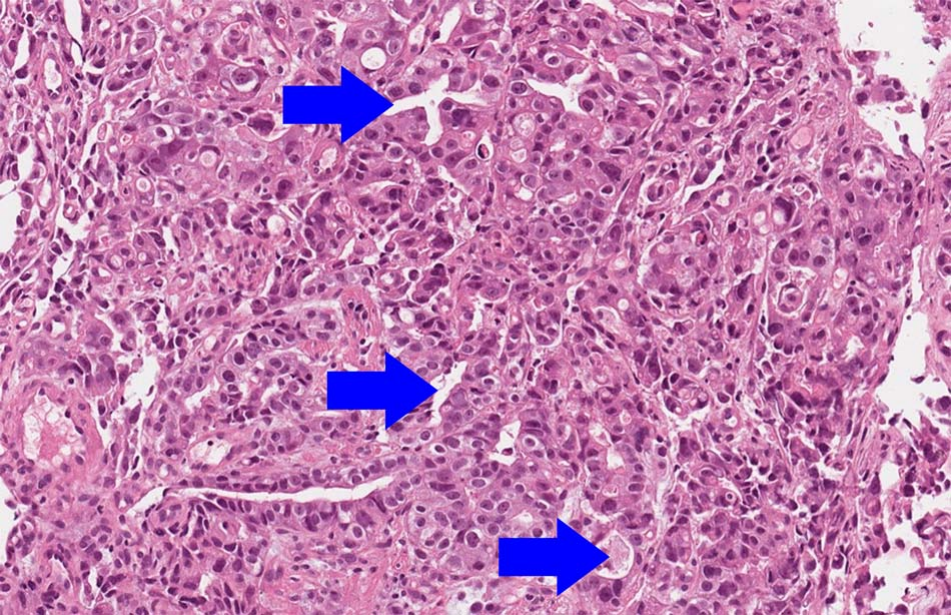
Biopsy (hematoxylin & eosin, 10× magnification): focal gland formation (blue arrows).

**Figure 4:**
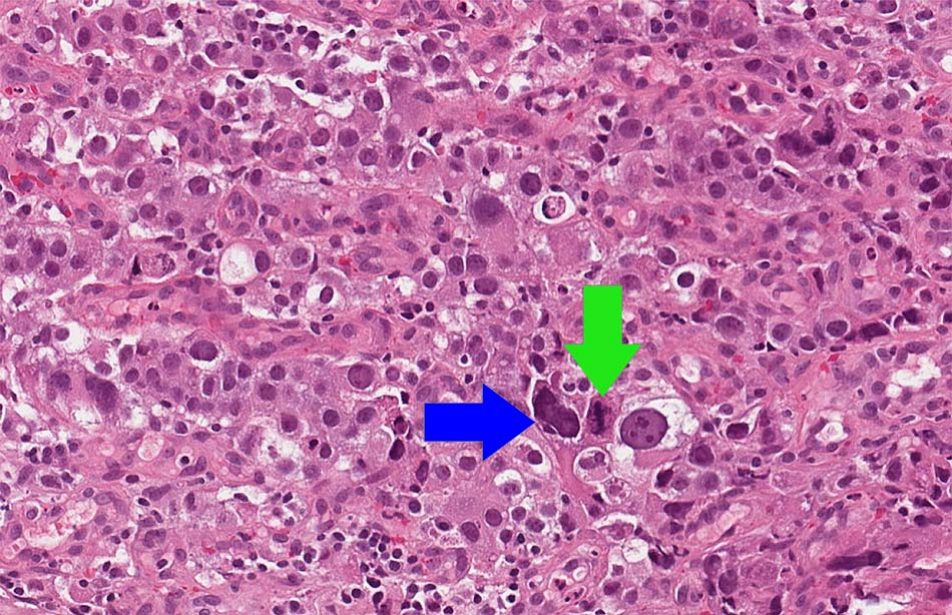
Biopsy (hematoxylin & eosin, 15× magnification): solid sheets of large polygonal cells with focal nuclear pleomorphism (blue arrow) and mitoses (green arrow).

**Figure 5:**
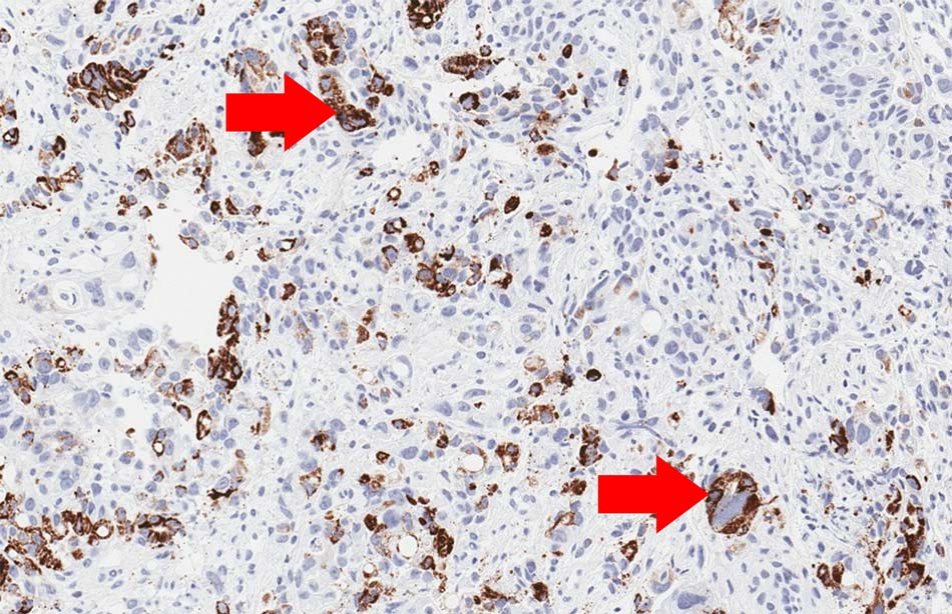
Biopsy (Hep-Par-1 immunohistochemistry, 10× magnification): focal positive cytoplasmic reaction (red arrows).

**Figure 6:**
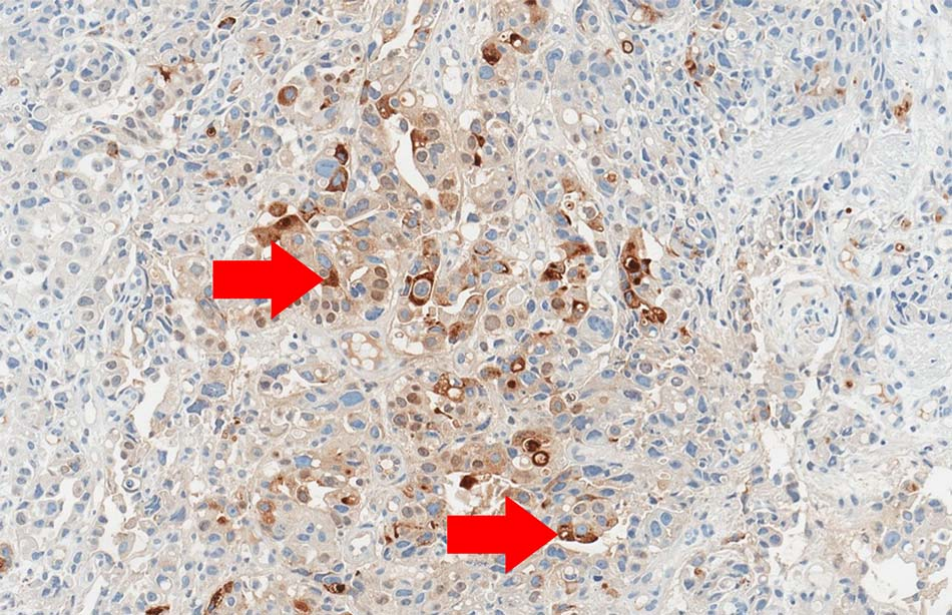
Biopsy (Alpha-fetoprotein (AFP) immunohistochemistry, 10× magnification): focal positive cytoplasmic reaction (red arrows).

Biopsy revealed an ulcerated tumor infiltrating lamina propria and the submucosal tissue (Figures 3 and 4). The tumor stained positively for cytokeratins (PanCK 100%/CK7 80%/CK20 30%), CDX2 (50%), HepPar-1 (15%) (Figure 5) and AFP (10%) (Figure 6). Positivity for E-cadherin, SMAD4, PMS2/MSH6 was retained, scattered Synaptophysin positive cells were seen. WT1, S100, HER2 reactions, *H. pylori* test were negative.

Tumor DNA was sequenced using AmpliSeq for Illumina Cancer Hotspot NGS panel. The data analysis revealed two somatic mutations in the *PIK3CA* gene: c.1023_1024insTACT (p.Thr342TyrfsTer12) and c.1025_1026insTA (p.Tyr343ThrfsTer3). Both mutations were located in exon 20 of *PIK3CA* gene and resulted in an early stop codon.

Due to heterogeneous histologic features and immunophenotype diagnosis clinicians were notified that this tumor fulfills the criteria of a very aggressive subtype, a hepatoid adenocarcinoma of the stomach (HAS).

After all the investigations, the 21-week-pregnant patient was diagnosed with stage IV gastric cancer (cT4 N1 M1). The case was discussed during a tumor board meeting. It was agreed that patient could only be treated with palliative chemotherapy. The patient was informed about her prognosis: she decided to terminate the pregnancy and start the treatment. After the abortion patient received 1st cycle of first-line systemic chemotherapy with the FOLFOX4 regimen (oxaliplatin, leucovorin, 5-fluorouracil), but for the second cycle she arrived in significantly deteriorated physical condition. Progression of disease was confirmed: new metastases in the ovary, enlarged lymphadenopathy, progressing ascites, and pleural effusion. The patient developed small bowel obstruction, diffuse peritonitis and died 2 months after the initial disease presentation.

## DISCUSSION

HAS is a rare neoplasm and the annual incidence is approximately 0.58–0.83 cases per million people. Pregnancy-associated HAS is an extremely rare and aggressive subtype that accounts for < 1% of all gastric cancer. High serum AFP levels during pregnancy can mask the AFP production by the tumor, so suspicion of HAS may become a challenge for physicians [1–3].

AFP-producing gastric tumor was first reported in 1970 by Bourreille et al. The term ‘hepatoid adenocarcinoma’ was coined by Ishikura et al. in 1985. It can originate in various organs including the gastrointestinal tract, ovary, colon, lung, urinary bladder, pancreas, etc. but the stomach is the most common site [5, 6]. It has histologic features resembling a hepatocellular carcinoma and can be confirmed by several immunohistochemical markers such as AFP, Glypican-3, SALL4, and Hep-Par 1. According to the literature, the incidence of AFP producing gastric cancer is from 1.3% to 15%, but AFP negative gastric cancer cannot exclude the possibility of HAS [7–10].

HAS is a clinicopathological entity with its unique aggressive behavior and no proven effective systemic therapy [7–10].

The treatment of metastatic HAS (mHAS) remains even less elucidated. Cisplatin-based chemotherapy is the most efficient first-line systemic treatment. Some drugs (e.g. 5-fluorouracil, oxaliplatin, irinotecan, docetaxel, paclitaxel) have been used for the treatment of mHAS. There is no standard chemotherapy regimen for treatment during pregnancy. The use of chemotherapy during the first trimester of pregnancy is not recommended. The majority of patients presents with poor response to conventional chemotherapy. No specific driver gene mutations and biomarkers in HAS are known to provide effective therapies [7–9].

The patient presented in our case received a systemic platinum-based chemotherapy FOLFOX regimen that is a safe and effective treatment in advanced gastric cancer. However, this treatment did not stop the progress of HAS and an aggressive course of the disease was observed. The pathogenesis of pregnancy-related gastric cancer is an issue of debate but in multivariate analysis, pregnancy was not found to be an independent risk factor [1].

We performed molecular genetic testing of tumor DNA to select the best treatment option. Biomarkers such as PD-L1, MSI were negative, so the patient could not benefit from immunotherapy. Gene mutation analysis by NGS revealed mutations in the *PIK3CA* gene that could explain the exceptionally aggressive course of this disease.

*PIK3CA* gene mutation has been reported in 5% of cases in gastric adenocarcinoma and is significantly associated with a poor prognosis. The PI3K pathway plays a fundamental role in cell proliferation, differentiation, mortality, and survival. In addition, increased *PIK3CA* mRNA have been identified in gastric cancer lymph node metastases, suggesting that *PIK3CA* may be an indicator of gastric cancer metastasis. *PIK3CA* gene alterations are predicted to be oncogenic [10].

Our finding showed that *PIK3CA* gene mutation is observable in HAS and may be one of the mechanisms in activating the PI3K/AKT pathway, which can be associated with HAS cancerogenesis and with poor survival of HAS patients. In the future, the PI3K/AKT signaling pathway may be an effective target in HAS and gastric cancer.

This case represents the first report of pregnancy-associated hepatoid gastric cancer with *PIK3CA* gene mutations. It can provide further clues for the understanding of molecular features of HAS and may lead to further effective treatment options.

## References

[1] Niknam R, Haghighat S, Mokhtari M. The pregnancy-associated gastric cancer: a case report and review of the literature. J Obstet Gynaecol. 2020;20:1–2. 10.1080/01443615.2019.1694880 31955632

[2] Pacheco S, Norero E. The Rare and Challenging Presentation of Gastric Cancer during Pregnancy: A Report of Three Cases. J Gastric Cancer. 2016;16:271–6. 28053815 10.5230/jgc.2016.16.4.271 PMC5206319

[3] Gastric cancer during pregnancy: A report on 13 cases and review of the literature with focus on chemotherapy during pregnancy. Acta Obstet Gynecol Scand. 2020;99:79–88. 10.1111/aogs.13731. 31529466 PMC6972614

[4] Wang Y, Sun L, Li Z. Hepatoid adenocarcinoma of the stomach: a unique subgroup with distinct clinicopathological and molecular features. Gastric Cancer. 2019;22. 10.1007/s10120-019-00965-5 PMC6811386 30989433

[5] Su JS, Chen YT. Clinicopathological characteristics in the differential diagnosis of hepatoid adenocarcinoma: a literature review. World J Gastroenterol. 2013;19:321–7. 23372352 10.3748/wjg.v19.i3.321 PMC3554814

[6] Kishimoto T, Nagai Y, Kato K, et al. Hepatoid adenocarcinoma: a new clinicopathological entity and the hypotheses on carcinogenesis. Med Electron Microsc 2000;33:57–63. doi.org/10.1007/s007950070002. 11810459 10.1007/s007950070002

[7] Liu X, Cheng Y, Sheng W, et al. Clinicopathologic features and prognostic factors in alpha-fetoprotein-producing gastric cancers: analysis of 104 cases. *J Surg Oncol*. 2010;102:249–55. 10.1002/jso.21624. 20740583

[8] Yang J, Wang R, Zhang W, Zhuang W, Wang M, Tang C. Clinicopathological and prognostic characteristics of hepatoid adenocarcinoma of the stomach. *Gastroenterol Res Pract*. 2014;2014:140587. 10.1155/2014/140587. 24669215 PMC3942340

[9] Baek SK, Han S, Oh D, Im SA, Kim TY, Bang YJ. Clinicopathologic characteristics and treatment outcomes of hepatoid adenocarcinoma of the stomach, a rare but unique subtype of gastric cancer. *BMC Gastroenterol*. 2011;11:56. 10.1186/1471-230X-11-56. 21592404 PMC3136411

[10] Liu JF, Zhou XK, Chen JH, et al. Upregulation of PIK3CA promotes metastasis in gastric carcinoma. World J. Gastroenterol. 2010; ePub Oct 2010. 10.3748/wjg.v16.i39.4986 PMC2957609 20954287

